# 1,2-Di-2-quinolylethene

**DOI:** 10.1107/S160053680900186X

**Published:** 2009-01-23

**Authors:** Hoong-Kun Fun, Reza Kia, Annada C. Maity, Rinku Chakrabarty, Shyamaprosad Goswami

**Affiliations:** a X-ray Crystallography Unit, School of Physics, Universiti Sains Malaysia, 11800 USM, Penang, Malaysia; b Department of Chemistry, Bengal Engineering and Science University, Shibpur, Howrah 711 103, India

## Abstract

The title compound, C_20_H_14_N_2_, comprises two crystallographically independent centrosymmetric mol­ecules (*A* and *B*) with different conformations due to the disorder of molecule *B*. The whole of mol­ecule *B* is disordered over two sets of positions, corresponding to a 180° rotation of the molecule, with a site-occupancy ratio of 0.780 (6):0.220 (6). The minor component of the disordered part in *B* has the same configuration as mol­ecule *A*, but the major component is different. The dihedral angle between the planes of mol­ecule *A* and mol­ecule *B* (major component) is 63.22 (3)°. The crystal structure is stabilized by inter­molecular C—H⋯π inter­actions.

## Related literature

For the biological activities, mol­ecular recognition and catalysis see, for example: Fournet *et al.* (2003 ▶); Yamada *et al.*, (1981 ▶); Goswami & Mahapatra (1998 ▶); Goswami *et al.* (1989 ▶).
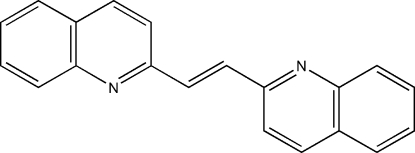


## Experimental

### 

#### Crystal data

C_20_H_14_N_2_
*M* *_r_* = 282.33
Monoclinic, 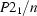

*a* = 15.6378 (2) Å
*b* = 6.0798 (1) Å
*c* = 16.0860 (2) Å
β = 108.879 (1)°
*V* = 1447.10 (4) Å^3^
*Z* = 4
Mo *K*α radiation
μ = 0.08 mm^−1^
*T* = 100.0 (1) K
0.34 × 0.33 × 0.09 mm

#### Data collection

Bruker APEXII CCD area-detector diffractometer
Absorption correction: multi-scan (**SADABS**; Bruker, 2005 ▶) *T*_min_ = 0.863, *T*_max_ = 0.993
12910 measured reflections
3317 independent reflections
2476 reflections with *I* > 2σ(*I*)
*R*_int_ = 0.030

#### Refinement

*R*[*F* ^2^ > 2σ(*F* ^2^)] = 0.040
*wR*(*F* ^2^) = 0.102
*S* = 1.04
3317 reflections
245 parameters
H-atom parameters constrained
Δρ_max_ = 0.25 e Å^−3^
Δρ_min_ = −0.17 e Å^−3^

### 

Data collection: *APEX2* (Bruker, 2005 ▶); cell refinement: *SAINT* (Bruker, 2005 ▶); data reduction: *SAINT*; program(s) used to solve structure: *SHELXTL* (Sheldrick, 2008 ▶); program(s) used to refine structure: *SHELXTL*; molecular graphics: *SHELXTL*; software used to prepare material for publication: *SHELXTL* and *PLATON* (Spek, 2003 ▶).

## Supplementary Material

Crystal structure: contains datablocks global, I. DOI: 10.1107/S160053680900186X/pk2144sup1.cif

Structure factors: contains datablocks I. DOI: 10.1107/S160053680900186X/pk2144Isup2.hkl

Additional supplementary materials:  crystallographic information; 3D view; checkCIF report

## Figures and Tables

**Table 1:** Hydrogen-bond geometry (Å, °)

*D*—H⋯*A*	*D*—H	H⋯*A*	*D*⋯*A*	*D*—H⋯*A*
C2*A*—H2*AA*⋯*Cg*1^i^	0.93	2.77	3.3409 (14)	121
C6*A*—H6*AA*⋯*Cg*2^ii^	0.93	2.65	3.5328 (18)	159
C4*B*—H4*B*⋯*Cg*3^iii^	0.93	2.85	3.376 (12)	116
C6*A*—H6*AA*⋯*Cg*3^iv^	0.93	2.76	3.613 (10)	152

Symmetry codes: (i) 
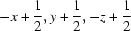
; (ii) 
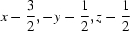
; (iii) 
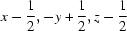
; (iv) 
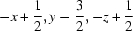
. *Cg*1, *Cg*2 and *Cg*3 are the centroids of the C3*A*–C8*A*, N1*B*/C8*B*/C3*B*/C2*B*/C1*B*/C9*B* and N1*C*/C8*C*/C3*C*/C2*C*/C1*C*/C9*C* rings, respectively.

**Fig. 1.**
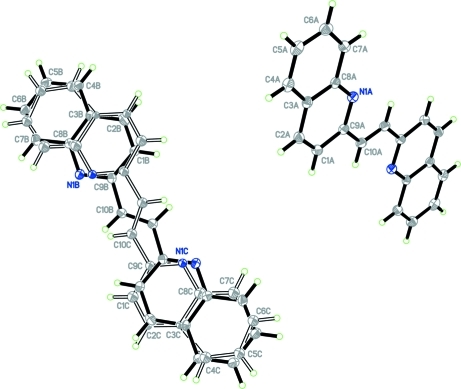
The molecular structure showing 40% probability displacement ellipsoids and the atomic numbering. Open bonds indicate the minor component [symmetry code for C: -x + 1, -y + 2, -z and symmetry code for unlabelled atoms -x, -y, -z].

**Fig. 2.**
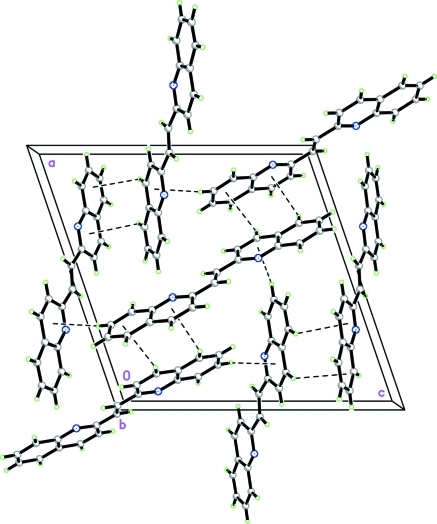
Crystal packing of viewed down the b-axis showing linking of molecules by intermolecular C—H···π interactions. Inermolecular interactions are drawn as dashed lines. Only the major component of the disordered molecule is shown.

## supplementary crystallographic information

### Comment

Alkene or alkyne substituted quinolines are important as they exhibit significant activities against HTLV-1 transformed cells and also show the efficiency of these compounds for the treatment of ATLL (Fournet *et al.,* 2003).

The benzylic carbon-carbon coupling reactions of benzylic halides catalyzed by Co^I^(PPh_3_)_3_Cl and also the synthesis of diaryl ethylene have been reported (Yamada *et al.,* 1981). The same reaction of functionalised benzylic bromides was shown to be useful for carbon-carbon bond formation by Co^I^ in the absence of oxygen, resulting in the convenient synthesis of a variety of functionalized benzylic dimers suitable for new spacers in molecular recognition research (Goswami & Mahapatra 1998; Goswami *et al.,* 1989). We report here a useful and straightforward procedure for the synthesis of 1,2-di-(2-quinolyl)-ethylene from 2,2-dichloromethyl quinoline.

The title compound, Fig. 1, comprises two crystallographically independent centrosymmetric molecules with different  conformations due to the disorder over two sites, corresponding to a *ca*180° rotation about the C9B—C10B bond. The minor component of the disordered part in B has the same configuration as molecule A, but the major component is different. The difference in conformation is that the A molecule atoms N1A-C9A-C10A-C10AA (AA is the symmetry related of A), form a chain like *U* shape while the corresponding atoms in the major component of B form a *Z* shape.   The dihedral angle between the plane of molecule A and molecule B is 63.22 (3)°. In molecule B, the whole molecule is disordered over two positions with a site-occupancy factor of 0.780 (6)/0.220 (6). The crystal structure is stabilized by intermolecular C—H···π interactions (C2A—H2AA···Cg1^i^, C6A—H6AA···Cg2^ii^, C4B—H4B···Cg3^iii^, and C6A—H6AA···Cg3^iv^: (i) 1/2 - *X*, 1/2 + *Y*, 1/2 - *Z*; (ii) -1/2 + *X*, 1/2 - *Y*, 1/2 + *Z*; (iii) 1/2 + *X*, 3/2 - *Y*, 1/2 + *Z*; (iv) 1/2 - *X*, -3/2 + *Y*, 1/2 - *Z*; *Cg*1, *Cg*2 and *Cg*3 are the centroids of the C3A–C8A, N1B/C8B/C3B/C2B/C1B/C9B and N1C/C8C/C3C/C2C/C1C/C9C aromatic rings).

### Experimental

2,2-dichloromethyl quinoline (1 mmol) was dissolved in dry benzene (25 mL). The anhydrous green colored Co^I^(PPh_3_)_3_Cl (2.5 mmol) catalyst was added to the reaction mixture with stirring at room temperature under a nitrogen atmosphere. After 30 minutes, the color of the reaction mixture had changed from green to blue. The reaction mixture was then heated under reflux conditions for 2-3 h. The solvent was evaporated to dryness, the residue was worked up with water and the organic part was extracted with chloroform. The organic layer was dried (Na_2_SO_4_) and concentrated. Column chromatography of the crude product on silica gel and elution with methanol in chloroform afforded 1,2-di-(2-quinolyl)-ethylene. Single crystals suitable for *X*-ray diffraction were grown by slow evaporation of a CHCl_3_-methanol (1:1) solution of the title compound.

### Refinement

All of the hydrogen atoms were positioned geometrically and constrained to refine with the parent atoms with C—H = 0.96 Å and  U_iso_ (H) = 1.2 U_eq_ (C). The whole molecule B is disordered by a 180° rotation over two positions with a site- occupancy factor of 0.780 (6)/0.220 (6). For the minor component, only isotropic refinement was used. Initially rigid, similarity and simulation restraints were applied to molecule B. After steady state has been reached, these restraints were removed for the final refinement. There is no restraint used in the final refinement.

### Crystal data

**Table:**

C_20_H_14_N_2_	*F*(000) = 592
*M**_r_* = 282.33	*D*_x_ = 1.296 Mg m^−^^3^
Monoclinic, *P*2_1_/*n*	Mo *K*α radiation, λ = 0.71073 Å
Hall symbol: -P 2yn	Cell parameters from 3767 reflections
*a* = 15.6378 (2) Å	θ = 2.7–31.5°
*b* = 6.0798 (1) Å	µ = 0.08 mm^−^^1^
*c* = 16.0860 (2) Å	*T* = 100 K
β = 108.879 (1)°	Block, yellow
*V* = 1447.10 (4) Å^3^	0.34 × 0.33 × 0.09 mm
*Z* = 4	

### Data collection

**Table:**

Bruker APEXII CCD area-detector diffractometer	3317 independent reflections
Radiation source: fine-focus sealed tube	2476 reflections with *I* > 2σ(*I*)
graphite	*R*_int_ = 0.030
φ and ω scans	θ_max_ = 27.5°, θ_min_ = 1.6°
Absorption correction: multi-scan (*SADABS*; Bruker, 2005)	*h* = −20→20
*T*_min_ = 0.863, *T*_max_ = 0.993	*k* = −7→6
12910 measured reflections	*l* = −20→18

### Refinement

**Table:**

Refinement on *F*^2^	Secondary atom site location: difference Fourier map
Least-squares matrix: full	Hydrogen site location: inferred from neighbouring sites
*R*[*F*^2^ > 2σ(*F*^2^)] = 0.040	H-atom parameters constrained
*wR*(*F*^2^) = 0.102	*w* = 1/[σ^2^(*F*_o_^2^) + (0.0416*P*)^2^ + 0.3849*P*] where *P* = (*F*_o_^2^ + 2*F*_c_^2^)/3
*S* = 1.04	(Δ/σ)_max_ < 0.001
3317 reflections	Δρ_max_ = 0.25 e Å^−^^3^
245 parameters	Δρ_min_ = −0.17 e Å^−^^3^
0 restraints	Extinction correction: *SHELXTL* (Sheldrick, 2008), Fc^*^=kFc[1+0.001xFc^2^λ^3^/sin(2θ)]^-1/4^
Primary atom site location: structure-invariant direct methods	Extinction coefficient: 0.0035 (10)

### Special details

**Table:**

Experimental. The low-temperature data was collected with the Oxford Cyrosystem Cobra low-temperature attachment.
Geometry. All esds (except the esd in the dihedral angle between two l.s. planes) are estimated using the full covariance matrix. The cell esds are taken into account individually in the estimation of esds in distances, angles and torsion angles; correlations between esds in cell parameters are only used when they are defined by crystal symmetry. An approximate (isotropic) treatment of cell esds is used for estimating esds involving l.s. planes.
Refinement. Refinement of F^2^ against ALL reflections. The weighted R-factor wR and goodness of fit S are based on F^2^, conventional R-factors R are based on F, with F set to zero for negative F^2^. The threshold expression of F^2^ > 2sigma(F^2^) is used only for calculating R-factors(gt) etc. and is not relevant to the choice of reflections for refinement. R-factors based on F^2^ are statistically about twice as large as those based on F, and R- factors based on ALL data will be even larger.

### Fractional atomic coordinates and isotropic or equivalent isotropic displacement parameters (Å^2^)

**Table:**

	*x*	*y*	*z*	*U*_iso_*/*U*_eq_	Occ. (<1)
N1A	0.07166 (7)	0.04930 (18)	0.16586 (7)	0.0216 (3)	
C1A	0.10529 (8)	0.3931 (2)	0.10857 (9)	0.0246 (3)	
H1AA	0.1003	0.4797	0.0596	0.030*	
C2A	0.14983 (8)	0.4706 (2)	0.19043 (9)	0.0247 (3)	
H2AA	0.1749	0.6108	0.1977	0.030*	
C3A	0.15781 (8)	0.3371 (2)	0.26439 (8)	0.0215 (3)	
C4A	0.20530 (8)	0.4010 (2)	0.35202 (9)	0.0263 (3)	
H4AA	0.2318	0.5395	0.3631	0.032*	
C5A	0.21245 (8)	0.2605 (2)	0.42038 (9)	0.0281 (3)	
H5AA	0.2442	0.3033	0.4776	0.034*	
C6A	0.17178 (9)	0.0509 (2)	0.40428 (9)	0.0281 (3)	
H6AA	0.1770	−0.0437	0.4511	0.034*	
C7A	0.12485 (8)	−0.0149 (2)	0.32079 (9)	0.0248 (3)	
H7AA	0.0977	−0.1528	0.3114	0.030*	
C8A	0.11711 (8)	0.1247 (2)	0.24836 (8)	0.0201 (3)	
C9A	0.06639 (8)	0.1799 (2)	0.09801 (8)	0.0218 (3)	
C10A	0.01868 (8)	0.0993 (2)	0.00925 (8)	0.0232 (3)	
H10A	0.0142	0.1934	−0.0375	0.028*	
N1B	0.69538 (18)	0.8831 (3)	0.07847 (10)	0.0206 (5)	0.780 (6)
C1B	0.57855 (14)	0.6355 (5)	0.08990 (15)	0.0210 (5)	0.780 (6)
H1B	0.5174	0.6104	0.0801	0.025*	0.780 (6)
C2B	0.6415 (3)	0.4802 (7)	0.1311 (3)	0.0247 (9)	0.780 (6)
H2B	0.6231	0.3476	0.1486	0.030*	0.780 (6)
C3B	0.7367 (3)	0.5221 (6)	0.1476 (2)	0.0161 (7)	0.780 (6)
C4B	0.8065 (3)	0.3683 (7)	0.1894 (3)	0.0215 (9)	0.780 (6)
H4B	0.7918	0.2321	0.2072	0.026*	0.780 (6)
C5B	0.89117 (17)	0.4192 (6)	0.20252 (17)	0.0241 (6)	0.780 (6)
H5B	0.9360	0.3167	0.2284	0.029*	0.780 (6)
C6B	0.91586 (16)	0.6283 (5)	0.17789 (16)	0.0236 (6)	0.780 (6)
H6B	0.9765	0.6623	0.1891	0.028*	0.780 (6)
C7B	0.8505 (2)	0.7811 (4)	0.13753 (16)	0.0219 (5)	0.780 (6)
H7B	0.8670	0.9174	0.1213	0.026*	0.780 (6)
C8B	0.7575 (3)	0.7292 (7)	0.1208 (2)	0.0182 (7)	0.780 (6)
C9B	0.6082 (2)	0.8361 (4)	0.06227 (11)	0.0192 (5)	0.780 (6)
C10B	0.54504 (13)	1.0039 (3)	0.01229 (11)	0.0208 (6)	0.780 (6)
H10B	0.5703	1.1285	−0.0039	0.025*	0.780 (6)
N1C	0.3376 (6)	1.1411 (12)	−0.0755 (4)	0.0140 (16)*	0.220 (6)
C1C	0.4292 (7)	1.4289 (18)	−0.1047 (6)	0.026 (2)*	0.220 (6)
H1C	0.4870	1.4748	−0.1013	0.031*	0.220 (6)
C2C	0.3606 (11)	1.556 (3)	−0.1416 (11)	0.014 (3)*	0.220 (6)
H2C	0.3687	1.6944	−0.1628	0.017*	0.220 (6)
C3C	0.2813 (9)	1.486 (2)	−0.1479 (10)	0.011 (3)*	0.220 (6)
C4C	0.2057 (13)	1.604 (3)	−0.1817 (12)	0.024 (4)*	0.220 (6)
H4C	0.2120	1.7485	−0.1976	0.029*	0.220 (6)
C5C	0.1046 (7)	1.515 (2)	−0.1969 (7)	0.020 (3)*	0.220 (6)
H5C	0.0529	1.5947	−0.2263	0.025*	0.220 (6)
C6C	0.1025 (8)	1.3099 (17)	−0.1622 (6)	0.022 (2)*	0.220 (6)
H6C	0.0474	1.2482	−0.1645	0.026*	0.220 (6)
C7C	0.1792 (8)	1.197 (2)	−0.1248 (6)	0.026 (3)*	0.220 (6)
H7C	0.1743	1.0569	−0.1035	0.031*	0.220 (6)
C8C	0.2620 (12)	1.271 (4)	−0.1160 (13)	0.030 (5)*	0.220 (6)
C9C	0.4178 (6)	1.2225 (17)	−0.0697 (5)	0.0172 (18)*	0.220 (6)
C10C	0.4987 (5)	1.0882 (12)	−0.0238 (4)	0.023 (2)*	0.220 (6)
H10C	0.5533	1.1342	−0.0294	0.028*	0.220 (6)

### Atomic displacement parameters (Å^2^)

**Table:**

	*U*^11^	*U*^22^	*U*^33^	*U*^12^	*U*^13^	*U*^23^
N1A	0.0207 (5)	0.0220 (6)	0.0214 (6)	0.0007 (4)	0.0058 (4)	−0.0004 (5)
C1A	0.0220 (6)	0.0249 (7)	0.0272 (7)	0.0016 (5)	0.0083 (5)	0.0051 (6)
C2A	0.0214 (6)	0.0195 (7)	0.0330 (8)	−0.0017 (5)	0.0084 (6)	−0.0009 (6)
C3A	0.0174 (6)	0.0220 (7)	0.0253 (7)	0.0022 (5)	0.0072 (5)	−0.0030 (5)
C4A	0.0227 (6)	0.0248 (7)	0.0301 (8)	0.0003 (5)	0.0068 (5)	−0.0085 (6)
C5A	0.0248 (7)	0.0365 (8)	0.0213 (7)	0.0032 (6)	0.0052 (5)	−0.0089 (6)
C6A	0.0282 (7)	0.0344 (8)	0.0222 (7)	0.0036 (6)	0.0088 (5)	0.0023 (6)
C7A	0.0250 (7)	0.0245 (7)	0.0251 (7)	−0.0009 (5)	0.0083 (5)	0.0005 (6)
C8A	0.0163 (6)	0.0215 (7)	0.0226 (7)	0.0012 (5)	0.0064 (5)	−0.0013 (5)
C9A	0.0189 (6)	0.0225 (7)	0.0236 (7)	0.0022 (5)	0.0065 (5)	0.0017 (5)
C10A	0.0214 (6)	0.0265 (7)	0.0209 (7)	0.0029 (5)	0.0058 (5)	0.0034 (6)
N1B	0.0168 (11)	0.0220 (9)	0.0217 (8)	−0.0001 (7)	0.0045 (7)	0.0001 (6)
C1B	0.0193 (9)	0.0226 (15)	0.0203 (10)	−0.0011 (9)	0.0051 (7)	−0.0014 (10)
C2B	0.0308 (15)	0.0216 (19)	0.0228 (16)	−0.0083 (12)	0.0103 (11)	−0.0013 (13)
C3B	0.0110 (15)	0.0222 (13)	0.0160 (11)	−0.0003 (12)	0.0056 (11)	−0.0020 (7)
C4B	0.0253 (17)	0.0185 (15)	0.0189 (14)	0.0117 (12)	0.0048 (11)	0.0069 (10)
C5B	0.0255 (12)	0.0219 (15)	0.0244 (11)	0.0049 (11)	0.0074 (8)	0.0034 (11)
C6B	0.0194 (11)	0.0273 (14)	0.0233 (11)	0.0025 (11)	0.0057 (8)	0.0023 (10)
C7B	0.0179 (13)	0.0234 (11)	0.0258 (11)	−0.0020 (11)	0.0089 (10)	−0.0010 (9)
C8B	0.022 (2)	0.0188 (13)	0.0133 (12)	0.0004 (14)	0.0051 (12)	−0.0022 (7)
C9B	0.0188 (11)	0.0192 (11)	0.0195 (9)	−0.0017 (9)	0.0061 (8)	−0.0012 (7)
C10B	0.0214 (11)	0.0190 (10)	0.0214 (9)	−0.0012 (7)	0.0061 (7)	0.0009 (7)

### Geometric parameters (Å, °)

**Table:**

N1A—C9A	1.3307 (16)	C4B—H4B	0.9300
N1A—C8A	1.3661 (15)	C5B—C6B	1.422 (4)
C1A—C2A	1.3592 (18)	C5B—H5B	0.9300
C1A—C9A	1.4183 (18)	C6B—C7B	1.378 (3)
C1A—H1AA	0.9300	C6B—H6B	0.9300
C2A—C3A	1.4118 (18)	C7B—C8B	1.426 (5)
C2A—H2AA	0.9300	C7B—H7B	0.9300
C3A—C4A	1.4183 (17)	C9B—C10B	1.466 (3)
C3A—C8A	1.4259 (18)	C10B—C10B^ii^	1.335 (4)
C4A—C5A	1.3683 (19)	C10B—H10B	0.9300
C4A—H4AA	0.9300	N1C—C9C	1.324 (9)
C5A—C6A	1.411 (2)	N1C—C8C	1.40 (2)
C5A—H5AA	0.9300	C1C—C2C	1.30 (2)
C6A—C7A	1.3652 (18)	C1C—C9C	1.410 (10)
C6A—H6AA	0.9300	C1C—H1C	0.9300
C7A—C8A	1.4146 (18)	C2C—C3C	1.28 (2)
C7A—H7AA	0.9300	C2C—H2C	0.9300
C9A—C10A	1.4650 (17)	C3C—C4C	1.34 (2)
C10A—C10A^i^	1.332 (3)	C3C—C8C	1.47 (3)
C10A—H10A	0.9300	C4C—C5C	1.61 (2)
N1B—C9B	1.333 (3)	C4C—H4C	0.9300
N1B—C8B	1.362 (5)	C5C—C6C	1.371 (13)
C1B—C2B	1.370 (5)	C5C—H5C	0.9300
C1B—C9B	1.426 (3)	C6C—C7C	1.344 (11)
C1B—H1B	0.9300	C6C—H6C	0.9300
C2B—C3B	1.448 (6)	C7C—C8C	1.335 (18)
C2B—H2B	0.9300	C7C—H7C	0.9300
C3B—C8B	1.403 (6)	C9C—C10C	1.486 (11)
C3B—C4B	1.429 (5)	C10C—C10C^ii^	1.311 (15)
C4B—C5B	1.308 (6)	C10C—H10C	0.9300
			
C9A—N1A—C8A	118.11 (11)	C7B—C6B—C5B	120.5 (2)
C2A—C1A—C9A	119.88 (12)	C7B—C6B—H6B	119.8
C2A—C1A—H1AA	120.1	C5B—C6B—H6B	119.8
C9A—C1A—H1AA	120.1	C6B—C7B—C8B	119.7 (2)
C1A—C2A—C3A	119.68 (12)	C6B—C7B—H7B	120.2
C1A—C2A—H2AA	120.2	C8B—C7B—H7B	120.2
C3A—C2A—H2AA	120.2	N1B—C8B—C3B	124.8 (4)
C2A—C3A—C4A	123.73 (12)	N1B—C8B—C7B	117.5 (3)
C2A—C3A—C8A	117.09 (11)	C3B—C8B—C7B	117.7 (3)
C4A—C3A—C8A	119.16 (12)	N1B—C9B—C1B	122.53 (17)
C5A—C4A—C3A	120.54 (13)	N1B—C9B—C10B	115.0 (2)
C5A—C4A—H4AA	119.7	C1B—C9B—C10B	122.4 (2)
C3A—C4A—H4AA	119.7	C10B^ii^—C10B—C9B	126.7 (2)
C4A—C5A—C6A	120.12 (12)	C10B^ii^—C10B—H10B	116.7
C4A—C5A—H5AA	119.9	C9B—C10B—H10B	116.7
C6A—C5A—H5AA	119.9	C9C—N1C—C8C	117.5 (11)
C7A—C6A—C5A	120.80 (13)	C2C—C1C—C9C	121.4 (10)
C7A—C6A—H6AA	119.6	C2C—C1C—H1C	119.3
C5A—C6A—H6AA	119.6	C9C—C1C—H1C	119.3
C6A—C7A—C8A	120.67 (13)	C3C—C2C—C1C	118.0 (14)
C6A—C7A—H7AA	119.7	C3C—C2C—H2C	121.0
C8A—C7A—H7AA	119.7	C1C—C2C—H2C	121.0
N1A—C8A—C7A	118.54 (12)	C2C—C3C—C4C	123.7 (16)
N1A—C8A—C3A	122.77 (12)	C2C—C3C—C8C	124.9 (14)
C7A—C8A—C3A	118.69 (11)	C4C—C3C—C8C	111.4 (14)
N1A—C9A—C1A	122.47 (12)	C3C—C4C—C5C	125.1 (14)
N1A—C9A—C10A	118.44 (12)	C3C—C4C—H4C	117.5
C1A—C9A—C10A	119.09 (12)	C5C—C4C—H4C	117.5
C10A^i^—C10A—C9A	124.71 (15)	C6C—C5C—C4C	113.2 (10)
C10A^i^—C10A—H10A	117.6	C6C—C5C—H5C	123.4
C9A—C10A—H10A	117.6	C4C—C5C—H5C	123.4
C9B—N1B—C8B	118.0 (3)	C7C—C6C—C5C	120.8 (11)
C2B—C1B—C9B	119.0 (2)	C7C—C6C—H6C	119.6
C2B—C1B—H1B	120.5	C5C—C6C—H6C	119.6
C9B—C1B—H1B	120.5	C8C—C7C—C6C	124.7 (14)
C1B—C2B—C3B	120.2 (3)	C8C—C7C—H7C	117.7
C1B—C2B—H2B	119.9	C6C—C7C—H7C	117.7
C3B—C2B—H2B	119.9	C7C—C8C—N1C	120.2 (18)
C8B—C3B—C4B	120.9 (4)	C7C—C8C—C3C	124.4 (16)
C8B—C3B—C2B	115.4 (4)	N1C—C8C—C3C	115.3 (14)
C4B—C3B—C2B	123.7 (4)	N1C—C9C—C1C	122.9 (8)
C5B—C4B—C3B	120.0 (4)	N1C—C9C—C10C	117.7 (9)
C5B—C4B—H4B	120.0	C1C—C9C—C10C	119.3 (9)
C3B—C4B—H4B	120.0	C10C^ii^—C10C—C9C	126.9 (9)
C4B—C5B—C6B	121.2 (3)	C10C^ii^—C10C—H10C	116.6
C4B—C5B—H5B	119.4	C9C—C10C—H10C	116.6
C6B—C5B—H5B	119.4		
			
C9A—C1A—C2A—C3A	0.64 (18)	C2B—C3B—C8B—N1B	−2.9 (5)
C1A—C2A—C3A—C4A	178.00 (12)	C4B—C3B—C8B—C7B	−0.6 (5)
C1A—C2A—C3A—C8A	−0.46 (17)	C2B—C3B—C8B—C7B	178.5 (3)
C2A—C3A—C4A—C5A	−178.12 (12)	C6B—C7B—C8B—N1B	−178.1 (2)
C8A—C3A—C4A—C5A	0.32 (18)	C6B—C7B—C8B—C3B	0.6 (4)
C3A—C4A—C5A—C6A	−0.58 (19)	C8B—N1B—C9B—C1B	2.0 (3)
C4A—C5A—C6A—C7A	−0.06 (19)	C8B—N1B—C9B—C10B	−176.8 (2)
C5A—C6A—C7A—C8A	0.96 (19)	C2B—C1B—C9B—N1B	−3.1 (3)
C9A—N1A—C8A—C7A	−178.59 (11)	C2B—C1B—C9B—C10B	175.6 (3)
C9A—N1A—C8A—C3A	0.75 (17)	N1B—C9B—C10B—C10B^ii^	179.9 (2)
C6A—C7A—C8A—N1A	178.17 (11)	C1B—C9B—C10B—C10B^ii^	1.1 (3)
C6A—C7A—C8A—C3A	−1.20 (18)	C9C—C1C—C2C—C3C	−2(2)
C2A—C3A—C8A—N1A	−0.24 (17)	C1C—C2C—C3C—C4C	177.6 (16)
C4A—C3A—C8A—N1A	−178.78 (11)	C1C—C2C—C3C—C8C	0(3)
C2A—C3A—C8A—C7A	179.10 (11)	C2C—C3C—C4C—C5C	175.5 (15)
C4A—C3A—C8A—C7A	0.56 (17)	C8C—C3C—C4C—C5C	−7(2)
C8A—N1A—C9A—C1A	−0.58 (17)	C3C—C4C—C5C—C6C	7(2)
C8A—N1A—C9A—C10A	179.29 (10)	C4C—C5C—C6C—C7C	−3.8 (16)
C2A—C1A—C9A—N1A	−0.11 (19)	C5C—C6C—C7C—C8C	2(2)
C2A—C1A—C9A—C10A	−179.98 (11)	C6C—C7C—C8C—N1C	179.5 (11)
N1A—C9A—C10A—C10A^i^	−1.6 (2)	C6C—C7C—C8C—C3C	−1(3)
C1A—C9A—C10A—C10A^i^	178.32 (15)	C9C—N1C—C8C—C7C	179.2 (13)
C9B—C1B—C2B—C3B	1.1 (5)	C9C—N1C—C8C—C3C	0.2 (19)
C1B—C2B—C3B—C8B	1.6 (6)	C2C—C3C—C8C—C7C	−178.3 (17)
C1B—C2B—C3B—C4B	−179.3 (4)	C4C—C3C—C8C—C7C	4(3)
C8B—C3B—C4B—C5B	−0.5 (6)	C2C—C3C—C8C—N1C	1(2)
C2B—C3B—C4B—C5B	−179.5 (4)	C4C—C3C—C8C—N1C	−176.8 (15)
C3B—C4B—C5B—C6B	1.6 (6)	C8C—N1C—C9C—C1C	−2.0 (15)
C4B—C5B—C6B—C7B	−1.5 (4)	C8C—N1C—C9C—C10C	177.9 (10)
C5B—C6B—C7B—C8B	0.3 (4)	C2C—C1C—C9C—N1C	3.2 (16)
C9B—N1B—C8B—C3B	1.1 (4)	C2C—C1C—C9C—C10C	−176.7 (11)
C9B—N1B—C8B—C7B	179.8 (2)	N1C—C9C—C10C—C10C^ii^	−11.3 (12)
C4B—C3B—C8B—N1B	178.1 (3)	C1C—C9C—C10C—C10C^ii^	168.6 (9)

Symmetry codes: (i) −*x*, −*y*, −*z*; (ii) −*x*+1, −*y*+2, −*z*.

### Hydrogen-bond geometry (Å, °)

**Table:**

*D*—H···*A*	*D*—H	H···*A*	*D*···*A*	*D*—H···*A*
C2A—H2AA···Cg1^iii^	0.93	2.77	3.3409 (14)	121
C6A—H6AA···Cg2^iv^	0.93	2.65	3.5328 (18)	159
C4B—H4B···Cg3^v^	0.93	2.85	3.376 (12)	116
C6A—H6AA···Cg3^vi^	0.93	2.76	3.613 (10)	152

Symmetry codes: (iii) −*x*+1/2, *y*+1/2, −*z*+1/2; (iv) *x*−3/2, −*y*−1/2, *z*−1/2; (v) *x*−1/2, −*y*+1/2, *z*−1/2; (vi) −*x*+1/2, *y*−3/2, −*z*+1/2.
